# AntiGQ1b antibody positive with MFS/GBS overlapped syndrome with diplopia and hemiplegia onset: Case report and retrospective analysis

**DOI:** 10.1097/MD.0000000000030584

**Published:** 2022-09-16

**Authors:** Mingmin Zhao, Yuxuan Gu, Jingru Zhao, Na Li

**Affiliations:** a Department of Neurology, Hebei General Hospital, Shijiazhuang, Hebei, China; b Graduate School of Hebei North University, Zhangjiakou, Hebei, China.

**Keywords:** ataxia, hemiplegia, MFS overlapped with GBS, antiGQ1B antibody, diplopia

## Abstract

GBS and MFS have been divided into several subtypes, constituting a series of independent and overlapping syndromes that share similar pathophysiology, leading to common clinical features, including history of previous infection, single-phase course, symmetry, skull or limbs weakness, CFS albumin cell separation (high protein, normal cell count), antiganglioside antibodies and axon, or evidence of demyelinating neuropathy neurophysiology. Part of the MFS in patients with clinical manifestations may be complicated, and even symptoms are not typical. A few patients may overlap with BBE or GBS.

**Patient concerns:**

Most patients with MFS/GBS overlap syndrome have a good prognosis, and a few patients may experience fluctuations or re-exacerbations. In most patients, after treatment, their neurological function basically recovers within a few weeks or months.

**Diagnosis interventions:**

The patient had ophthalmoplegia, ataxia, weak force, and protein-cell separation in cerebrospinal fluid during the development of the disease. The diagnosis of MFS overlapped with typical GBS was considered. The CSF specific IgG oligoclonal zone and anti-Sulfatide antibody were positive. Anti-GT1a IgG was positive. Anti-GQ1b IgG was positive, which supported the diagnosis of GBS spectrum disorders. According to their common immunological basis, plasma exchange or intravenous immunoglobulin (IVIG) therapy is recommended, which can effectively improve the symptoms and shorten the course of the disease.

**Outcomes:**

After treatment with glucocorticoids and gamma globulin, the symptoms improved and the patient was discharged.

**Lessons:**

MFS/GBS Superimposed syndrome is a rare clinical disease. Therefore, more attention should be paid to early diagnosis and treatment of similar patients to avoid misdiagnosis. Cerebral spinal fluid (CFS) examination, neuroelectrophysiology, and GQ1b antibody detection can be used to confirm the diagnosis.

## 1. Introduction

Miller Fisher syndrome (MFS) is a variant of Guillain-Barre syndrome (GBS), which is a relatively rare autoimmune disease. Typical clinical manifestations include the classic triad of ophthalmoplegia, ataxia, and areflexia.^[1]^ GBS and MFS have many subtypes, forming a continuous spectrum of discrete and overlapping syndromes. They have similar pathophysiological mechanisms, resulting in common clinical characteristics, including a history of antecedent infection, a monophasic disease course, symmetrical cranial or limb weakness, albuminocytological dissociation (high protein, normal cell count) in CFS, antiganglioside antibodies, and neurophysiological evidence of axonal or demyelinating neuropathy. The clinical manifestations of BBE and GBS can be complex and variable in some patients; thus, the clinical diagnosis and treatment of similar patients should focus on making an early diagnosis to prevent misdiagnosis. Cerebral spinal fluid examination, neuronal electrophysiology, GQ1b antibody detection, and other auxiliary examinations can be used to support the ultimate diagnosis. This article reports a relatively rare case of MFS/GBS overlapped syndrome with diplopia and hemiplegia, thus making a retrospective analysis of relevant antibodies combined with the literature to further understand the syndrome.

## 2. Case presentation

Two weeks prior to hospitalization, a 20-year-old man had cold. He presented with diplopia and gait instability on the right side, which was characterized by vertigo and rotation. The patient developed decreased flexibility of the right limb and felt it was difficult to hold on to the right upper extremity. The rotation was notable if the gaze was oriented to the right. The patient was hospitalized in November 2021. On the sixth day after hospitalization, the patient had limited abduction and adduction of the right eye and limited adduction of the left eye. Physical examination: The main positive signs of the patient were as follows: Abduction of the right eye was slightly limited, without eye movement deficits in the remaining directions. An eye gaze on both sides can lead to horizontal nystagmus, and an upward gaze can lead to vertical nystagmus. The right limb muscle strength was grade IV. The alternate right motion was inflexible. The right finger-to-nose test result was unstable. The heel knee-shin test result was impaired on the right side. Romberg’s sign is positive. The bilateral knee tendons were active. The patient’s right Babinski sign was positive. Tandem walking test results were strongly positive. Examination after admission includes blood analysis; blood homocysteine determination; biochemistry; coagulation function; rheumatic related examination; thyroid function tests; vasculitis screening; C-reactive protein + alpha fetoprotein + CA199 + CEA + human immunodeficiency virus + treponema pallidum; urine analysis; stool analysis; electrocardiograph; carotid ultrasound; lower limbs ultrasound; chest computed tomography(CT); cranial CT and cranial magnetic resonance imaging(MRI): normal.Ten tips for serum virus: herpes simplex virus I (IgG):387.383AU/mL, Rubella virus IgG:44.181IU/mL, Cytomegalovirus -IgG:425.183AU/mcg. Electromyography(EMG) suggested that the N20 latency of the left median nerve was slightly longer than that of the contralateral median nerve, the amplitude of P100 was lower than that of the contralateral median nerve, and the latency of the left nerve was prolonged. Cerebral spinal fluid cytology was dominated by lymphocytic reactions. Autoimmune encephalitis antibody test results were negative; CFS-OB5 + 24 h intrasheath synthesis rate of IgG was positive (Table 1). Routine and biochemical tests of the cerebral spinal fluid indicated an increase in the WBC count (13 × 10^6^/L), while the others were normal (Table 2). Because of evidence of symptoms and signs involving the central nervous system, the patient was initially diagnosed with brainstem encephalitis: viral encephalitis; however, BBE refused immunoglobulin and was given symptomatic treatment such as ganciclovir (250 mg, q12 h) for antiviral and vertigo. After high-dose pulse therapy with methylprednisolone sodium succinate (1000 mg/day intravenous drip), the dose was reduced and changed to oral prednisone acetate tablets. His eyes moved freely in all directions, his right limb was significantly improved, and he was discharged. One month later, he experienced gait instability again, and cranial MRI findings were normal. The patient was hospitalized again, and cerebral spinal fluid tests were reexamined. This suggested albuminocytological dissociation(high protein, normal cell count) in CFS (Table 2). A series of autoimmune peripheral neuropathy antibodies, such as antiGT1a antibody, antiGQ1B antibody, and antisulfatide antibody were positive(Table 3). AntiGQ1b antibody syndrome, and gamma globulin was administered for static treatment. Glucocorticoid therapy was continued to improve, and the patient was discharged.

**Table 1:** Autoimmune encephalitis antibody and CFS-OB5 + 24 h IgG results.

Inspection	Results
NMDA-R-Ab	Negative
CASPR2-Ab	Negative
AMPA1-R-A	Negative
AMPA2-R-A	Negative
LGI1-Ab	Negative
GABAB-R-A	Negative
GAD65-Ab	Negative
OB (CSF)	Positive
Oligo-clonal zone of CSF IgGSOB (CSF) cerebrospinal fluid specific IgG oligo-clonal zone	Positive

**Table 2:** Routine and biochemistry of cerebrospinal fluid.

CSF data	2021-12-1	2022-1-20
Color	Colorless	Colorless
White blood cells	13 × 10^6^/L	5 × 10^6^/L
Protein characterization	Negative	Negative
Pan reaction test	Negative	Negative
Chlorine	126 mmol/L	The tendency for 127.1 L
Protein	0.18 g/L	1.56 g/L

**Table 3:** Autoimmune peripheral neuropathy antibodies results.

Antibody data	Results
Anti sulfatide IgG	Positive
Anti-gt1a IgG	Positive
Anti-gq1b IgG	Positive

## 3. Discussion

GBS is an inclusive term that describes a group of related diseases with varying presentations, including MFS and GBS subtypes.^[2]^ Common clinical features include a history of antecedent infection, a monophasic disease course, and symmetrical cranial or limb weakness. These clinical features reflect the shared pathophysiological mechanisms. Researchers subclassified GBS spectrum diseases into 2 types: GBS classic and GBS variants, GBS variants include Acute Motor Sensory Axonal Neuropathy, (AMSAN), Acute Sensory Neuropathy (ASN), Pharyngeal-cervical-brachial syndrome(PCB), MFS and BBE.^[3]^ However, some scholars have proposed that pure sensory GBS, pure sensory ataxia, and BBE are incomplete forms of MFS and/or variants of GBS, which is worthy of further research.^[4]^ GBS and MFS have been subclassified into several subtypes, which together form a continuous spectrum with discrete and overlapping syndromes^[5]^ involving cranial nerves and limbs. MFS is a major variant of GBS and is characterized by ophthalmoplegia, ataxia, and areflexia,^[6]^ without limb weakness. The patient had additional symptoms involving reticular formation, thereby profoundly affecting consciousness, and was considered to have the central nervous system (CNS) subtype, known as BBE.^[7]^ There are also several incomplete forms of MFS defined by symptom presentation, such as the presence or absence of ophthalmoplegia or ataxia. There is also overlap between the variants. Five percent of patients with MFS develop limb weakness during the disease, which is considered as MFS overlapping with typical GBS. GBS laboratory tests of CSF suggesting albuminocytological dissociation have shown that CSF proteins were increased in 49% of patients on day 1, 88% after 2 weeks, and 15% had mild pleocytosis (5–50 cells/mL) but not more than 50 cells/mL.^[8]^

In this case, the patient had a cold 2 weeks before hospitalization, with “ophthalmoplegia” as the first symptom, which gradually worsened. Physical examination revealed grade IV right-limb muscle strength. The alternate right motion was inflexible. The right finger-to-nose test result was unstable. The heel knee-shin test result was impaired on the right side. Romberg’s sign is positive. The bilateral knee tendons were active. The patient’s right Babinski sign was positive. Tandem walking test results were strongly positive. EMG suggested that the N20 latency of the left median nerve was slightly longer than that of the contralateral median nerve, the amplitude of P100 was lower than that of the contralateral median nerve, and the latency of the left nerve was prolonged. Cranial CT and magnetic MRI were normal. Leukocytosis was observed in the cerebral spinal fluid (CSF). It also suggested albuminocytological dissociation (high protein, normal cell count) in the CFS after 6 weeks. The patient developed ophthalmoplegia, characterized by ataxia and progressive weakness, suggesting albuminocytological dissociation (high protein, normal cell count) in the CFS. Therefore, the diagnosis of MFS overlapped with the classical GBS.

GBS not only affects peripheral nerves; approximately 10% of patients show normal or hyperexcitable deep tendon reflexes throughout the illness.^[8,9]^ The Babinski sign positivity, disturbance of consciousness, and active deep tendon reflexes have been used to distinguish between MFS and BBE. Although recent studies have shown that symptoms of central nervous system involvement may be present in MFS patients, the brainstem and cerebellum are the most common sites of central nervous system involvement in MFS. Kim et al^[10]^ confirmed that patients with MFS had abnormal changes in glucose metabolism in the cerebellum and brainstem. Sandler Robert et al^[11]^ further verified that magnetic resonance spectroscopy (MRS) in MFS patients with negative cranial MRI identified a significant reduction in the N-acetyl aspartate/creatine (NAA/Cr) ratio in both the vermis and right hemisphere. This finding suggests cerebellar dysfunction. When the NAA/Cr ratio returned to normal after 2.5 months, clinical symptoms also recovered at the same time. In addition, individual patients with MFS may have evidence of white matter involvement. Xu et al^[12]^ reported the case of a male patient with MFS with unilateral and large lesions in the cerebral white matter. The affected sites included the juxtacortex, subcortex, and deep white matter, but the brainstem and cerebellum were not involved. The affected sites showed inflammatory demyelinating changes. He also had no symptoms of central nervous system involvement, such as reduced consciousness or seizure. These studies indicate that the central nervous system is involved in the occurrence and development of MFS. Therefore, patients with typical GBS or MFS characteristics may have positive pathological signs and normal or even active tendon reflexes, while tendon reflexes may also be reduced in the early stages of BBE. Hence, a positive Babinski sign and active tendon reflexes cannot be used as diagnostic criteria to exclude the possibility of MFS. In the diagnostic criteria considered by the GBS expert group in 2014, the presence or absence of consciousness disorders such as lethargy has been regarded as the main distinguishing point between MFS and BBE.^[2]^

This patient had diplopia with onset of hemiplegia, bilateral knee tendon reflexes, and a positive Babinski sign on the right side. At present, no similar case reports have been reported in clinical practice, suggesting that central nervous system involvement can be seen in typical GBS or MFS in the early stages.

Studies have shown that antiganglioside and antisulfatide antibodies are closely related to the pathogenesis of autoimmune-mediated acute and chronic polyneuropathy.^[13]^ Serum antiganglioside antibody tests can help to support the diagnosis of GBS.^[14]^ Gangliosides consist of a ceramide attached to 1 or more carbohydrates and sialic acid (N-acetylneuraminic acid) attached to an oligosaccharide core. GQ1b, GT1a, GD1a, GM1, etc. GS antibodies bind to gangliosides in peripheral nerves to activate complement, and the antibody and its complement-mediated immune complexes cause tissue damage. Furthermore, according to the specific distribution of each ganglioside in the body, different ganglioside antibody positivity has corresponding clinical manifestations. Microorganisms such as Campylobacter jejuni and Haemophilus influenzae, may induce antiGQ1b antibodies and interact with the extramedullary portion of the oculomotor, trochlear, and abducens nerves as well as intramuscular spindle class Ia afferent fibers in the limb and GQ1b antigen binding in the brainstem. This leads to a spectrum of the autoimmune continuum of disorders of the peripheral and central nervous systems.^[15]^ GQ1b is highly expressed in the extramedullary portion of the oculomotor, trochlear, abducens, glossopharyngeal, and vagus nerves and at the neuromuscular junction, as well as in-class Ia afferent fibers in the muscle spindle, and some large neurons in the dorsal root ganglia. Positive GQ1b antibodies can cause ophthalmoplegia and ataxia and can also bind to GQ1b expressed in the brainstem, resulting in consciousness disorder accompanied by a pyramid sign.^[16,17]^ Studies have shown a high positive rate of IgG antibodies to GQ1b in patients with MFS (83–95%) and BBE (66–68%).^[18]^ In 2013, Shahrizaila^[19]^et al proposed the concept of antiGQ1b antibody syndrome, with the deepening of the understanding of GQ1b antibody, and it is increasingly recognized that MFS and BBE are 2 ends of the antiGQ1b antibody syndrome continuum, and can manifest as various combinations of central and peripheral nervous system involvement. Antibodies against to GQ1b may explain the complex symptomatology and overlap between MFS, BBE, and GBS in this disease series, suggesting a common immunopathogenesis. (2)GT1a is more commonly expressed on the nerve membranes of the glossopharyngeal and vagal nerve.GT1a antibody involvement can lead to dysphagia, while PCB can lead to increased antiGT1a antibody.^[20]^ (3)Sulfatide is an acidic glycolipid and the main lipid component of myelin. It is synthesized in myelin-producing cells, oligodendrocytes of the central nervous system, and non-compact myelin Schwann cells in the peripheral nervous system. It also exists in the nodes and paranode areas of Langfield, accounting for about 4–7% of all myelin lipids. It plays an important role in maintaining the structure of the nerve sheath membrane, regulating nerve impulses, and transmitting membrane information.^[13]^ Campagnolo et al believed that screening with antisulfatide antibodies can be used as the basis for the diagnosis of peripheral neuropathy.^[21]^ Jiangxi Provincial Children’s Hospital also suggested that antisulfatide antibodies are involved in the pathogenesis of GBS spectrum diseases through a related study of 8 cases of antisulfatide antibody-positive Guillain-Barré syndrome.^[22]^

The patient was positive for cerebral spinal fluid-specific IgG oligoclonal zone. Among the 24 autoimmune peripheral neuropathy items, antisulfatide antibody IgG was positive, antiGT1a antibody IgG was positive, and antiGQ1b antibody IgG was positive, supporting the diagnosis of GBS spectrum diseases.

At present, there are no randomized controlled clinical trials on the treatment and intervention of MFS. If it has a common immunological basis with GBS, plasma exchange or intravenous immunoglobulin(IVIG) treatment is recommended, which can effectively improve symptoms and shorten the course of the disease. However a large retrospective study found that neither plasma exchange nor IVIG influenced patient outcomes, and new treatment options in the future may include complement inhibitor antibodies, and immunological therapies seem to be the direction of development.^[23]^Evidence for the use of steroids in the treatment of GBS patients is conflicting; its efficacy in antiGQ1b antibody syndrome is unclear, and it is difficult to distinguish its actual therapeutic effect from the process of disease self-healing. In particular, classic GBS has repeatedly demonstrated that steroid therapy is not only ineffective but also delays the resolution of clinical symptoms when combined with IVIG therapy.^[24]^ Given that the pathogenesis of GQ1b antibody syndrome is similar to that of GBS, it is not recommended unless used in combination with IVIG.

## Author contributions

All the authors contributed equally to this work. All authors have read and approved the final manuscript.

## Acknowledgment

The authors would like to thank Na Li for her assistance in writing this manuscript.
